# Multichannel mapping of in vivo rat uterine myometrium exhibits both high and low frequency electrical activity in non-pregnancy

**DOI:** 10.1038/s41598-024-57734-3

**Published:** 2024-03-27

**Authors:** Amy S. Garrett, Mathias W. Roesler, Omkar N. Athavale, Peng Du, Shawn A. Means, Alys R. Clark, Leo K. Cheng

**Affiliations:** Auckland Bioengineering Institute, University of Auckland, Auckland, New Zealand

**Keywords:** Uterus, Smooth muscle, Electrophysiology, In vivo, Spatial mapping, Biomedical engineering, Extracellular recording

## Abstract

The uterus exhibits intermittent electrophysiological activity in vivo. Although most active during labor, the non-pregnant uterus can exhibit activity of comparable magnitude to the early stages of labor. In this study, two types of flexible electrodes were utilized to measure the electrical activity of uterine smooth muscle in vivo in anesthetized, non-pregnant rats. Flexible printed circuit electrodes were placed on the serosal surface of the uterine horn of six anesthetized rats. Electrical activity was recorded for a duration of 20–30 min. Activity contained two components: high frequency activity (bursts) and an underlying low frequency ‘slow wave’ which occurred concurrently. These components had dominant frequencies of 6.82 ± 0.63 Hz for the burst frequency and 0.032 ± 0.0055 Hz for the slow wave frequency. There was a mean burst occurrence rate of 0.76 ± 0.23 bursts per minute and mean burst duration of 20.1 ± 6.5 s. The use of multiple high-resolution electrodes enabled 2D mapping of the initiation and propagation of activity along the uterine horn. This in vivo approach has the potential to provide the organ level detail to help interpret non-invasive body surface recordings.

## Introduction

The uterus is an electrically active smooth muscle organ. Although most active in labor, the non-gravid uterus is constantly electrically and mechanically active^1–4^. Wave-like activity in the myometrium varies in magnitude and frequency in response to changes in reproductive hormones^4^. In humans, the menstrual cycle is hallmarked by key events such as ovulation and menstruation, and contractions related to menstruation can be comparable in magnitude to that of early labor. The mechanisms which allow for healthy, non-pregnant contraction of the smooth muscle in the uterus are not well understood. Therefore, little is known about clinical disorders concerning the electrical activity of the uterus^5^. Abnormally painful period cramps and endometriosis (when the menstrual lining migrates to the abdominal cavity) are uterine smooth muscle pathologies which have significant impact on health and wellbeing^6–8^.

Animal models provide a useful avenue for invasive analysis of uterine activity. Rodents such as rats and mice have bicornuate uteri (two uterine horns in a Y-shaped structure) as opposed to the pyriform shape of the human uterus, and a 4–5 day hormonal cycle (as opposed to the 28 day human menstrual cycle)^9,10^. The myometrial layer of the uterus comprises smooth muscle cells in a complex structure. In rodents, the smooth muscle cells lie in two distinct layers, aligned in the longitudinal and circumferential directions, and there is some evidence of a bridging layer between the two. In humans, these layers are less distinct, and the composition appears more complex than can be divided into two specific layers^11^. Along with smooth muscle cells, both the rodent and human myometrium contains specialized neural cells called telocytes, also referred to as Interstitial Cajal-like Cells (ICLCs)^12,13^. These cells are so named for their similarity to the Interstitial Cells of Cajal (ICC) found in the smooth muscle layer of the gastrointestinal tract^14^. ICC in the stomach and small intestines initiate and coordinate electrical events key to peristalsis. However, the function of telocytes in uterine smooth muscle is not fully understood. When the behavior of telocytes is inhibited via disruption of the c-kit transmembrane receptor (found to be vital to maintain activity in the GI tract), the smooth muscle cells continued to spontaneously contract at the same frequency (although amplitude was effected at the highest concentration of antagonist)^15^, indicating these cells may not fulfill the pacing pacemaking role of ICCs in the stomach^16^. However, they may play a role in hormone sensing^17^. There is some evidence of initiation of uterine electrical activity at the proximal, ovarian end of the uterine horn in pregnant rats^18^, but there has been no comprehensive mapping of the origin of electrical activity to date, in pregnancy or non-pregnancy.

Bozler was the first to study the electrophysiological properties of the uterus by stimulating strips of myometrium from different animals outside of pregnancy^19^. Subsequently, many studies were conducted to understand the electrophysiological activity in the uterus of pregnant animals^1,20–22^. Recently, the use of high-resolution electrode arrays has led to a better understanding of the spatial patterns that occur and helped identify potential locations for the origin of the electrical activity^18,23^. Early in vivo studies on non-pregnant rats observed variations of the electrical activity by suturing electrodes to the myometrium and analyzing the duration and frequency of occurrence of bursts of electrical events^24,25^. Although they identified variations in these metrics, primarily in the amplitude and frequency of bursts, their results vary widely and there is little information on spatial propagation due to their use of limited number of electrodes. Peristaltic activity is crucial in the function of the non-pregnant uterus^26^ and propagates towards the cervix, although the direction of contraction can vary between primarily ovarian-cervical and cervical-ovarian^24,25^. A commonality in the literature when analyzing uterine electrophysiology is to consider primarily the high frequency activity. The analysis of ‘bursts’, or short sections of activity comprising many high frequency ‘spikes’ over a period of 5–20 s approximately, has typically been carried out to look at burst occurrence rate, burst duration etc. However, often the data is filtered to remove the low frequency information from the data^18,23,27–29^, not considering any slow wave components. Although there has been some limited investigation of slow waves in humans during late pregnancy and labor using non-invasive electrohysterograpy (EHG)^30–32^, little is known about both high frequency and low frequency electrophysiology outside of pregnancy.

In this study, flexible printed circuit (FPC) electrodes were used to map serosal electrical activity of the non-pregnant rat uterus in vivo. Similar FPC electrodes, previously developed and used to map gastrointestinal electrophysiology^33,34^, allow for the detection of the waves and bursts of electrical activity throughout the myometrium. Additionally, frequency analysis and spatial patterns of electrical activity can be quantified. This study provides a proof of concept for successful use of this methodology to investigate electrophysiology of the non-pregnant uterus and highlights the existence of slow waves which occur in concert with the bursts of fast, spiking activity reported previously. Furthermore, it enables the ability to directly measure the electrical signals of the uterus, and therefore aid in the development of non-invasive measurement techniques such as EHG.

## Methods

### Electrodes

**Figure 1:**
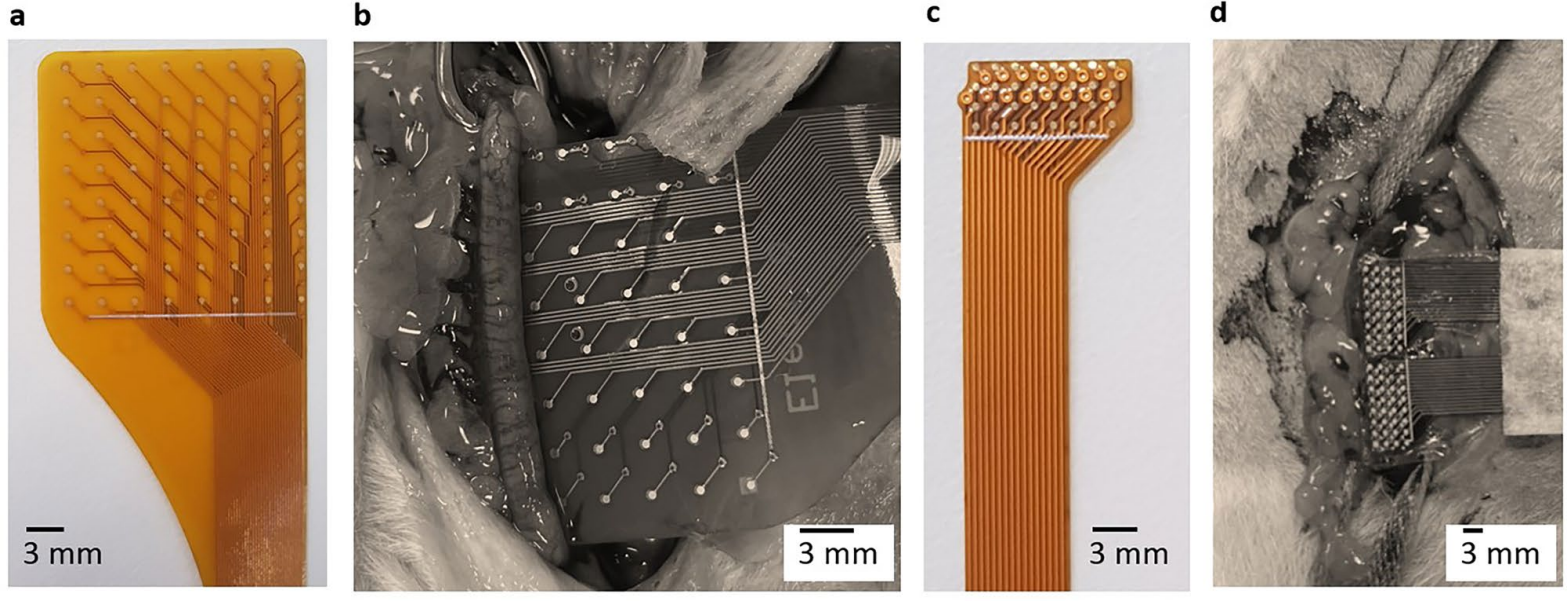
The two types of FPC electrodes used in the in vivo experiments, including E1 (**a**) and the E2 (**c**) and their placement above or below a horn of the uterus (**b**,**d**). E1 had an interelectrode distance of 3 mm in a 8 × 8 configuration, with size constraints allowing for at maximum one column of electrodes in contact with the uterine horn. E2 had a higher spatial resolution, with an interelectrode spacing of 0.95 mm in a 4 × 8 configuration. Multiple E2 electrodes can be used in combination, such as the two electrodes shown in d, to obtain a larger spatial coverage.

For this study, custom FPC electrode arrays were designed to perform high-resolution mapping of the electrical activity of the in vivo rat uterine horn. The electrodes were designed in Altium Designer 17.0 (Altium, La Jolla, Ca) and manufactured by PCBWay (Hangzhou, China). Two FPC electrode designs were used: electrode 1 (E1) and electrode 2 (E2). E1 was comprised of 64 electrodes in an 8 × 8 configuration with an electrode diameter of 0.51 mm and a regular interelectrode spacing of 3 mm^33,35^. Due to the anatomy of the rat uterus, only a single column of E1 electrodes was able to make contact with the uterine horn during experiments. To account for this limitation a second type of electrode was developed. Electrode E2 was comprised of a 4 × 8 electrode configuration with an electrode diameter of 0.38 mm and an interelectrode spacing of 0.95 mm. With E2, multiple columns of electrodes were able to maintain contact with the serosa of the uterus. To increase the area of measurement, multiple E2 arrays were used in combination for some studies. Figure 1 shows the E1 (A,B) and E2 (C,D) electrode configurations and their placement for in vivo experimentation (B and D). The electrical activity measured by each electrode was recorded using a BioSemi ActiveTwo (BioSemi, Amsterdam, the Netherlands) mapping system and analyzed using MATLAB (version 2023a, The Mathworks Inc.).

### Experimental procedure

Ethics approval was granted by the University of Auckland Animal Ethics Committee, and all experiments were carried out in accordance with the University of Auckland Animal Ethics guidelines and is reported in accordance with the ARRIVE guidelines. Virgin female Wistar or Sprague–Dawley rats were used in this study (n = 6, 291 ± 38 g). No specific estrus stage was selected for. Anesthesia was induced and maintained with 2–3% isoflurane in 100% oxygen throughout each experiment. Body temperature and heart rate were monitored and maintained within normal physiological limits. The rats were euthanized via cervical dislocation while under anesthesia at the completion of the study.

While under anesthesia, a midline laparotomy was performed to expose the uterus. The uterine horns were located and the FPC electrodes (Fig. 1) were carefully inserted beneath or on top of the uterine horn with minimal handling. Electrode E1 was used in the first 5 studies, and Electrode E2 was used in the last study. Where possible at least 1 row or column of electrodes were aligned along the length of the horn, to give spatial information about electrical activity. The abdomen was covered with plastic film to maintain heat and moisture during the recording. Bioelectric signals were recorded for approximately 30 min for each experiment.

### Data acquisition and analysis

Recordings were acquired at 512 Hz with a bandwidth of 0–400 Hz (DC coupled) and subsequently down sampled to 30 Hz for computational efficiency. Additional filtering was performed in software. A moving median filter with a window size of 10 s was used to provide an estimate the baseline drift. This estimate of the baseline drift was then subtracted from the original signal^36,37^. A notch filter was used to remove respiration artifact, which occurred at different frequencies between experiments within the range of 0.5–0.8 Hz, and the first two harmonics, if required. A randomly selected section of activity lasting approximately 500 s was isolated from each experimental recording to use for the analysis. A low pass filter (cut off frequency 1.5 Hz) was used to isolate the low frequency (slow wave) information from the signals. A high pass filter (1.5 Hz) was used to isolate the high frequency (burst and spike) activity.

Periods of activity were identified throughout the 500 s section of data. The occurrence rate of both the bursts and the slow waves was determined from this section of activity. The burst occurrence rate was defined as the number of burst sections of activity per minute and as determined by identifying the first spike in each burst event and defining their occurrence per minute of signal. The slow wave occurrence rate was similarly determined as the number of large depolarizations in the section of data. To assess the dominant frequency component of the burst and slow wave activity, the high- and low-pass filtered data was analyzed with a Fast Fourier transform (FFT), and the frequency with highest power determined. Burst duration was calculated as the time difference between the first spike and the last spike in the burst in each channel. Slow wave propagation velocity was determined as the distance a slow wave travelled along a line of electrodes, divided by the time. If the activity for a given experiment was disorganized, and there was no ordered propagation pattern, the propagation velocity was not calculated. A summary of the mean burst and slow wave frequencies, occurrence rates and burst durations for each of the n = 6 animals in the study is presented as the mean ± standard deviation (SD).

## Results

**Figure 2:**
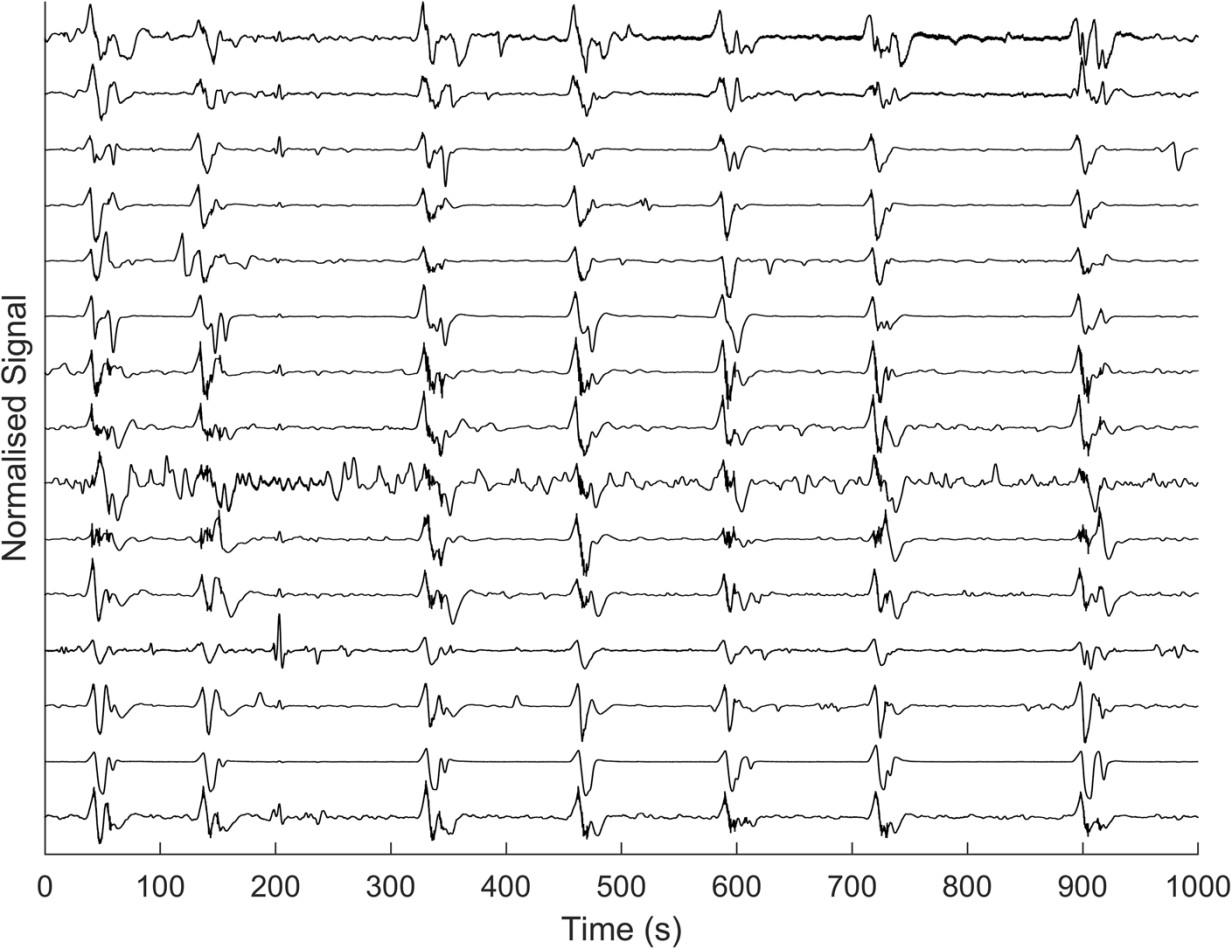
Example of in vivo electrophysiology data from a linear group of electrodes along a rat uterine horn, showcasing intermittent periods of activity at a rate of 0.42 bursts per minute (0.007 Hz) over a period of 16 min.

In all six animals, spontaneous intermittent electrical activity was recorded with the slow waves and spike bursts occurring concurrently (on a one-to-one basis). Figure 2 displays an example of typical signal traces obtained from the electrodes. This example data highlights the intermittent periods of activity with 7 cycles occurring over a 16 min period, therefore with an activity occurrence rate of 0.42 cycles per minute. These periods of activity contained both low frequency slow wave activity (the large magnitude depolarizations) simultaneous with the high frequency burst activity (comprising groups of individual spikes).

**Figure 3:**
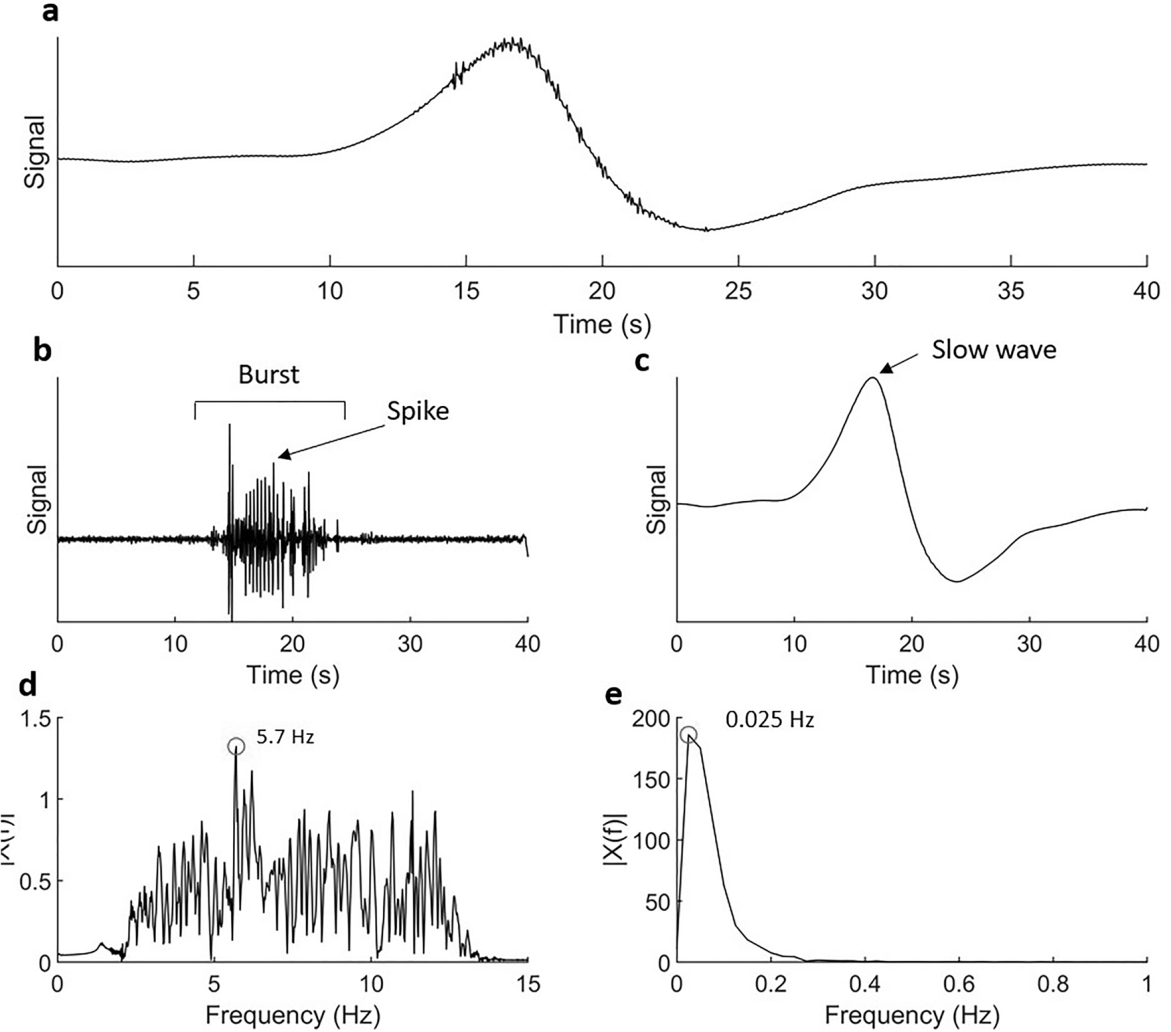
A 40 s section of electrophysiology data from one electrode (**a**) partitioned into high frequency (**b**) and low frequency (**c**) activity using a high pass (f = 1.5 Hz) and low pass (f = 1.5 Hz), filter respectively. The burst, comprised of multiple spikes, and the slow wave have been indicated on each respective plot. The FFT of a 500 s section of high frequency data (**d**) shows a dominant frequency component of the burst activity at 5.7 Hz and the corresponding low frequency activity (**e**) had a dominant frequency component of 0.025 Hz.

The electrophysiology data (a 40 s example shown in Fig. 3a) can be partitioned into high frequency (Fig. 3b) and low frequency (Fig. 3c) components. When analyzing the high frequency information, the sections of activity are identified as bursts, comprising multiple spikes. The FFT of the high frequency information (Fig. 3d) identifies the power associated with the frequency components of the burst data. For the example shown in Fig. 3, the dominant frequency component of the full 500 s dataset was 5.7 Hz. Similarly, the FFT of the 500 s slow wave data is shown in Fig. 3e, identifying the dominant slow wave frequency component of 0.025 Hz. The low frequency data exhibited a slow wave event followed by a distinct period of quiescence, resulting in the dominant slow wave frequency being different from the activity occurrence rate.

**Figure 4:**
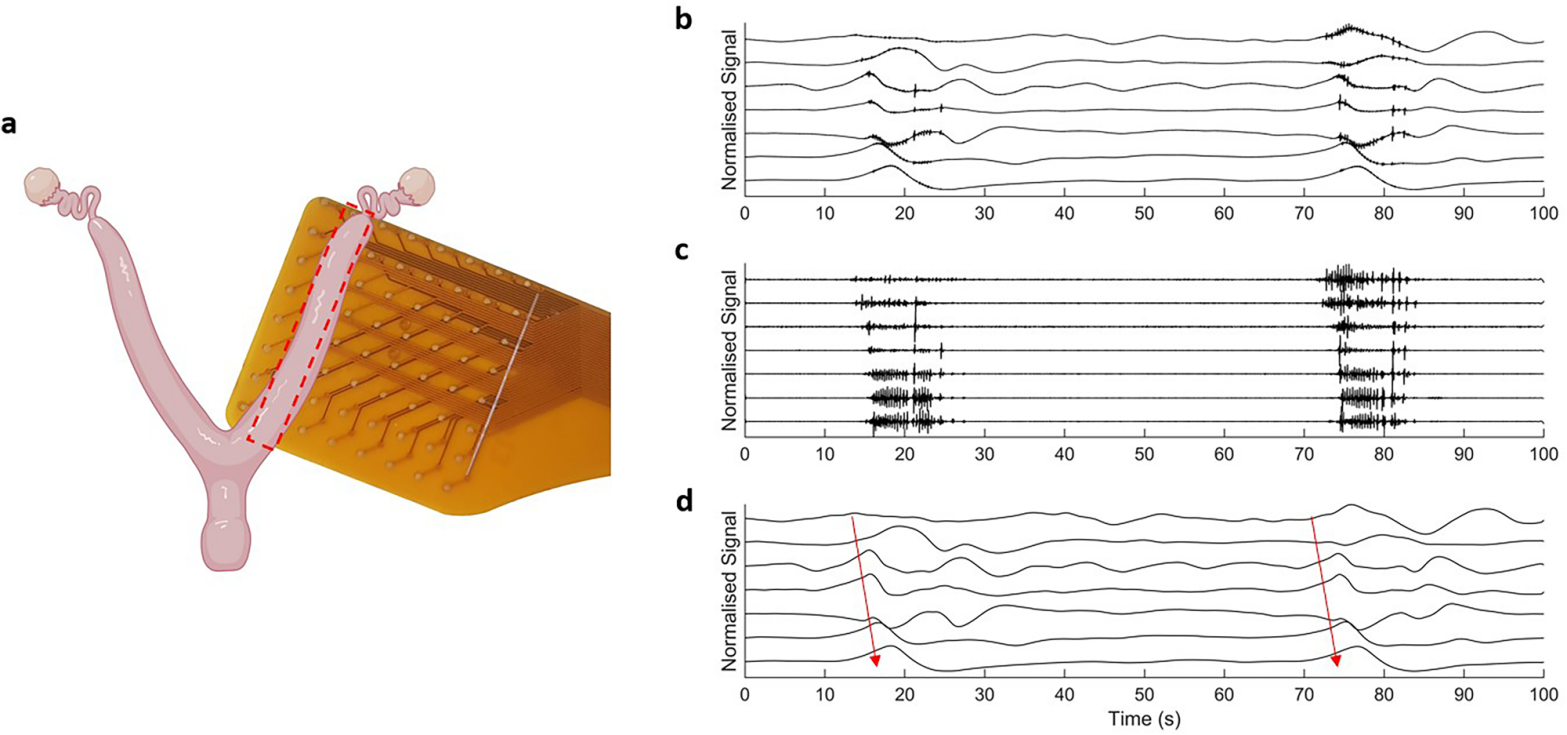
Electrical traces from the non-pregnant rat uterus using electrode E1. Seven adjacent electrodes were aligned with the horn, with an interelectrode spacing of 3 mm. Panel (**b**) shows electrode data with two cycles of activity occurring, approximately 60 s apart. Panel (**c**) shows high pass filtered data (cutoff frequency = 1.5 Hz). Panel (**d**) shows the low pass filtered data (cutoff frequency = 1.5 Hz) and the arrows indicate the direction of slow wave propagation. Panel (**a**) created with BioRender.com.

Figure 4 shows an example of a linear array of electrode data measured from the E1 electrode in vivo. Figure 4b shows an example of a burst of activity. This 100 s section of data has been filtered to remove any respiration artifact and the first two harmonics (using a second order digital notch filter at 0.74 Hz) and a moving median filter applied to remove baseline drift (window size of 10 s). Figure 4c displays the same signals when processed with a high pass filter (f = 1.5 Hz) to remove the slow wave activity and isolate only the spike activity. Figure 4d displays the signals once processed with a low pass filter, showing the slow wave activity in isolation. For this experiment the activity propagated from the ovarian to cervical end of the uterine horn at a speed of 5.37 ± 2.32 mm/s.

**Figure 5:**
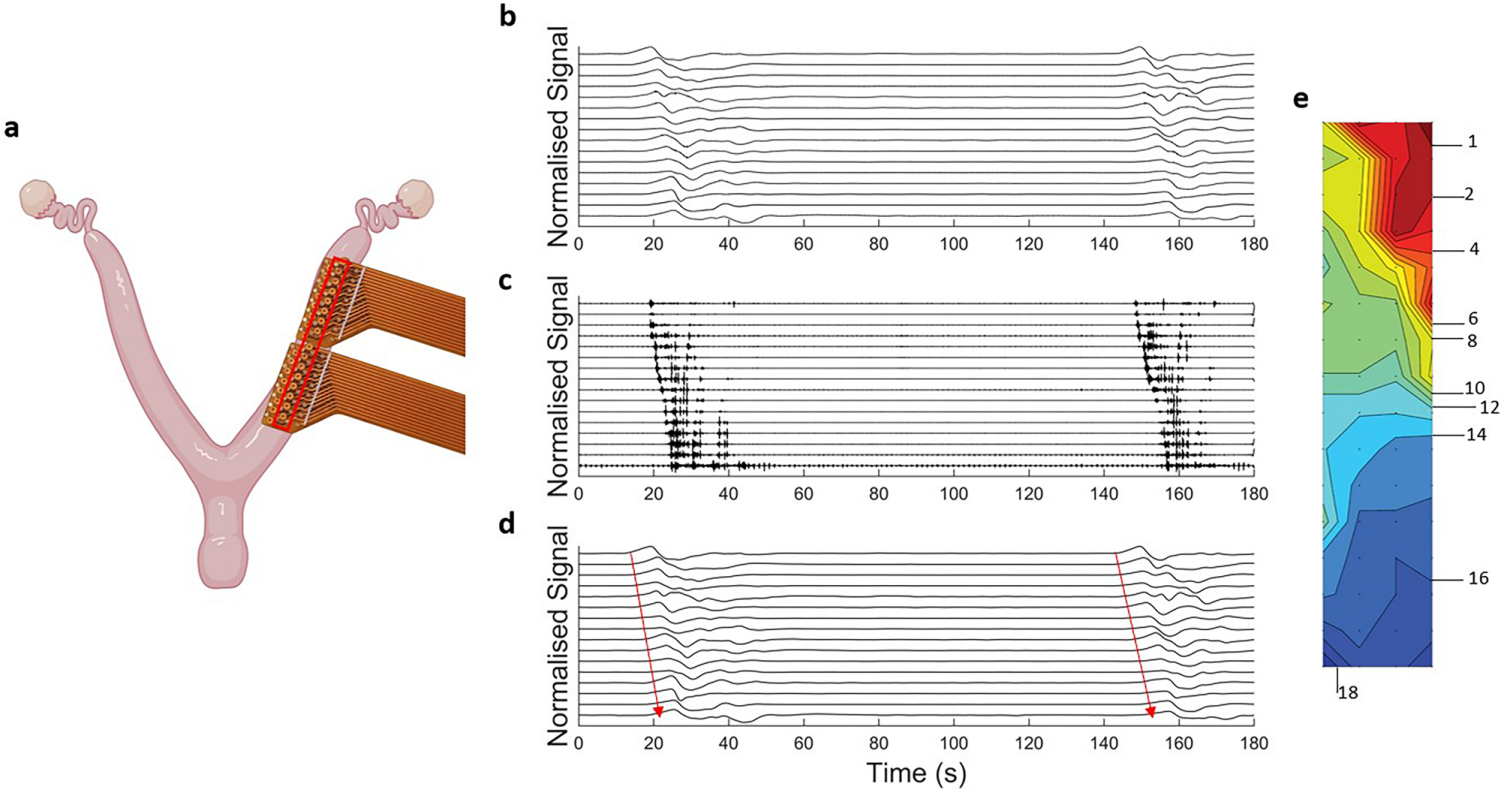
Electrical traces obtained from a non-pregnant rat uterus using electrode E2. (**a**) Placement of two high resolution electrodes. The 180 s section of activity from 16 electrodes (**b**) in a linear configuration along the centre of the array, outlined in red in panel (**a**), showing two cycles of activity separated by approximately 130 s (note the high-frequency activity is masked by the larger low-frequency components). The high pass and low pass components of the activity are shown in panels (**c**) and (**d**), respectively (cut off frequencies = 1.5 Hz for both). Arrows indicate the direction of propagation. The isochrone map of a burst of propagation is shown in panel (**e**), with each propagation line equal to 1 s. Panel (**a**) created with BioRender.com.

Figure 5 displays a 180 s subset of data from an experiment where two high resolution E2 electrodes were used in tandem, resulting in a 4 × 16 array of 64 electrodes in contact with the left uterine horn, as shown by the diagram in panel a. Panel b shows the data from 16 electrodes from along the length of the uterine horn, outlined in red. The high frequency bursts (Panel c) and the low frequency slow waves (Panel d) show the propogation of the bursts and slow wave from the ovarian end of the horn, towards the cervical end, indicated by the arrow in panel c. The increase in the spatial resolution of the E2 electrodes allows for more electrodes to be in contact with the uterine horn in vivo, and therefore 2-dimensional mapping can be achieved. The isochrone map for a burst of activity is shown in panel e, which each isochrone line representing 1 s of the propagation. For this experiment the activity propagated from the ovarian to cervical end of the uterine horn at 1.59 ± 0.37 mm/s.

**Figure 6:**
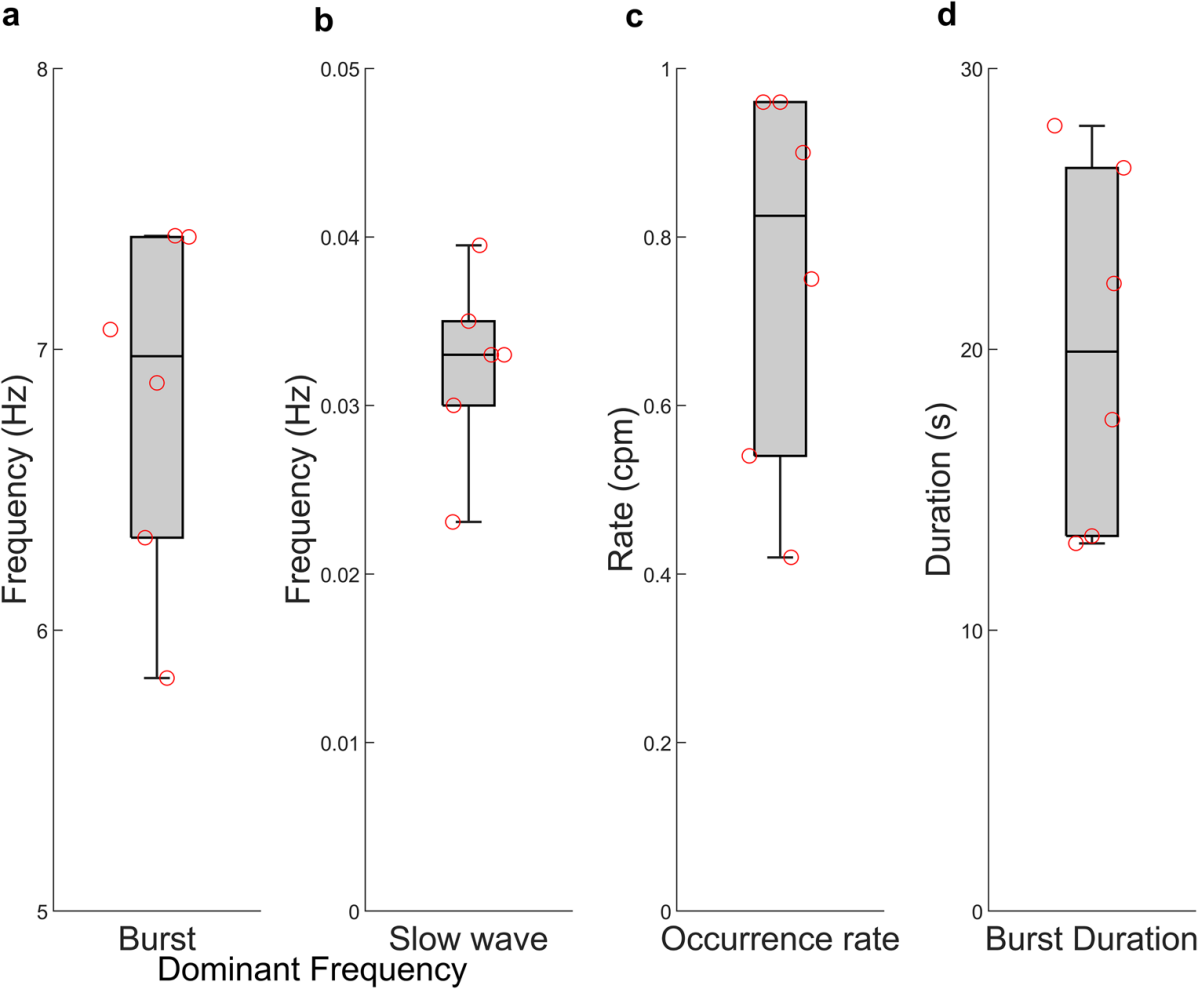
Characteristics of the electrical activity observed across six animals, showing (**a**) dominant burst frequency, (**b**) dominant slow wave frequency, (**c**) activity occurrence rate (for both slow waves and bursts) and (**d**) burst duration.

For the six animals included in this study, the dominant spike frequency, dominant slow wave frequency, burst occurrence rate, and burst durations is summarized in Fig. 6. Table 1 outlines the principle quantitative outputs from six rat experiments. The burst frequency is the dominant frequency component of the high pass filtered data (6.82 ± 0.63 Hz), and the slow wave frequency is the dominant frequency component of the low pass filtered data (0.032 ± 0.0055 Hz, or 1.9 ± 0.33 cycles per min). The burst and slow wave occurrence rate was determined (0.76 ± 0.23 per minute) and as the bursts and slow waves occurred simultaneously, the two occurrence rates were equal. The burst duration was calculated as 20.1 ± 6.5 s.

**Table 1:** Spike and slow wave frequency, burst occurrence rate and burst duration.

	Dominant frequency component (Hz)		Occurrence rate (burst per min)		Electrode used
	Bursts	Slow wave	Burst and slow wave	Burst duration (s)	
1	7.07	0.023	0.54	28.0	E1
2	6.33	0.030	0.96	13.1	E1
3	5.83	0.033	0.90	22.3	E1
4	7.40	0.033	0.75	26.5	E1
5	6.88	0.035	0.96	13.4	E1
6	7.40	0.040	0.42	17.5	E2
Mean ± SD	6.82 ± 0.63	0.032 ± 0.0055	0.76 ± 0.23	20.1 ± 6.5	

## Discussion

To measure, analyze and characterize the electrical activity of the non-pregnant rat uterus, we employed the use of flexible electrodes for in vivo measurement. Utilizing two electrode designs, electrophysiological signals were obtained from the serosal surface of the uterus, in rats under anesthesia. In all six animals, high and low frequency electrical activity generated by the myometrium was measured, and in experiments where electrodes were aligned along the length of the uterine horn, propagation velocity was determined. This provides the first in vivo measurements of uterine electrical activity characterized by high frequency spiking activity, along with a low frequency slow wave depolarization in non-pregnancy.

### Bursts and spikes

Although telocytes do not appear to act in a pacemaking fashion as ICCs do in the GI tract, they are present throughout the tissue, and some studies have hypothesized involvement with the initiation of electrical events^16^. Linking the electrical and contractile activity in uterine smooth muscle is the action of intracellular Ca^2+^^38^. Elevated Ca^2+^ levels driven by both fluxes through the plasma membrane of the cells and release from intracellular stores^22,39,40^ activate the smooth muscle contractile machinery, facilitating phosphorylation which forms cross-bridge links between actin and myosin kinases^41^. Unlike striated muscles, whose function critically depends on Ca^2+^ induced Ca^2+^ release from intracellular stores, uterine myometrial contractions instead respond primarily to Ca^2+^ influx via L-type voltage-gated channels on the plasma membrane^42^. Electrical depolarization propagating over the myometrial layers are key to predicting and tracking contractions of the uterine organ^20^. Hence, measuring electrical signals on the uterus are of great interest for monitoring and detecting deviations from healthy function.

Measurements of electrical activity have been carried out in a number of prior animal models, but these studies frequently employ ex vivo methods^18,23^. Lutton et al.^18^ and Lammers et al.^23^ showed only the high frequency spike activity, comparable to that shown in Figs. 3, 4 and 5. Their studies reported a burst occurrence rate of 1.0 ± 0.3 bursts per minute in pregnant rat^23^, and a burst duration of 39.2 ± 14.6 s^18^ in comparison to our non-pregnancy measurements of 0.76 ± 0.23 burst per minute and 20.1 ± 6.5 s duration. Although their experiments successfully recorded electrical activity, ex vivo experimentation could result in disruption to the inherent hormonal and neural signals which influence uterine activity. In addition, as these studies were carried out during late pregnancy, and there remains a lack of knowledge about the baseline electrical activity of the uterus in non-pregnancy. A limited number studies have been performed in vivo*,* however, they have employed low-resolution mapping techniques meaning spatial information cannot be obtained^24,28^, or employed alternate measurements of contraction activity such as image tracking^43^.

Ishikawa and Fuch observed, via the use of in vivo sutured electrodes in conscious animals along with an intrauterine pressure balloon, that spiking activity occurred simultaneously with an instance of a pressure wave in the intrauterine space^24^. This confirmed a correlation between these periods of intermittent activity and a global pressure wave within the intrauterine space. A similar approach by Talo et al. took a closer look at variations with estrus^25^. While these studies were ahead of their time for in vivo experiments, our study uses high resolution electrodes that improve the detail in electrical signal propagation that can be measured. Our novel in vivo measurement technique enables the possibility to maintain the blood supply and neural innervation of the tissue for the duration of the experiment, without the physical attachment of the electrodes to the tissue via suturing. Our measurements are comparable, both qualitatively and quantitatively, with previous observations in these low resolution in vivo sutured experiments, and with high-resolution ex vivo experiments in pregnant rats.

### Slow waves

Slow waves are crucial for the coordination of motility patterns in other smooth muscle organs, such as the stomach and small intestines^34,44^, but the characterization of such events in the uterus has not yet been achieved. In humans, global pressure waves within the intrauterine space have been recorded using pressure sensors, and the direction of contractions measured by abdominal ultrasound. Both spike activity and slow waves are likely to play a role in the local and global contraction activity in the uterus, previously described as ‘wave like’ patterns of relative quiescence in non-pregnancy, but of which the cycle-dependent patterns are vital for successful reproduction^4^. The alignment of spikes, slow waves and intrauterine pressure is important in continual development of non-invasive clinical measurement methods both during labor, and for diagnosis of clinical conditions^45,46^.

Experiments performed ex vivo have focused on high frequency signals, using bandpass filtering to remove low frequency noise and baseline drift. Previous investigators filtered between 2 and 400 Hz^18,23^ in the rat and between 5 and 50 Hz in pigs^27–29^ and examples of individual instances of spiking activity in the pregnant rat uterus showed a preference for propagation in the ovarian to cervical direction and a preference for initiation to occur at the mesometrial boarder^23^. Using our in vivo approach, we observed slow wave activity occurring simultaneously to the bursts of high frequency spike activity. In all cases, we observed bursts and slow waves occurring on a one-to-one basis, where every slow wave was associated with a spike burst. However, a previous study has reported changes in spike burst amplitude and frequency during different stages of the estrus cycle, and also that spike burst activity is limited or absent in the later part of diestrus and early part of the proestrus stages^25^. Therefore, although in this study there was a one-to-one correspondence between the two activities this may not always be the case. Our methods have the potential to provide further understand of the relationship between local high frequency electrical contractions, slower wave-like contractions throughout the uterine tissue, and the implications these contract types have on the intrauterine space and uterine function in non-pregnancy.

### Limitations and future applications

This study aimed to demonstrate feasibility of acquiring in vivo maps of uterine electrophysiological activity, along with characterization of slow waves from measurements of the serosal surface of the uterus. We have shown that slow wave and burst activity can be characterized with these methods. Although overall consistent measurements were obtained, there is some variability in the quantitative metrics characterizing electrical behaviors (Fig. 6, Table 1) which may be related to estrus. A limitation of the current study is the lack of estrus testing. In the future, by including estrus testing in our experimental protocol we aim to further characterize the behavior of the myometrium in response to hormonal changes. The utilization of high-resolution electrodes will provide the ability to map initiation and propagation of electrical waves throughout the uterine horn at different stages of the estrus cycle. Figure 3a shows the presence of some spike/burst activity. In the future it may be necessary to optimize or overlap the frequency bands of the high pass and low pass filters, to ensure no signal information is missed.

Another limitation of this method is that smooth muscle function may be affected by anesthesia. It has been found that gastrointestinal motility can be impaired by application of general anesthetics such as isoflurane, via disruption of cholinergic mechanisms^47,48^. Care should be taken when determining the manner of anesthesia for future experiments, and consideration as to possible effects of anesthesia on smooth muscle function, and how this may affect results.

Two types of electrodes (E1 and E2) were applied in this study. The relatively large inter-electrode spacing of E1 meant only a limited number of electrodes were able to be in contact with the electrodes, and importantly only a 1D representation of spatial activity. To address this limitation, an improved electrode E2 allowed for multiple rows of electrodes to be in contact with the uterus. The use of this electrode improved the reliability and accuracy of spatial characterization of propagation direction and speed. Another area of future analysis is comparing the timing of the slow waves and bursts, to determine if one type of activity precedes another.

The role of electrical and mechanical contraction in non-pregnancy is not well characterized. The predominant theory around the role of electrical activity is around ovulation, fertilization and implantation required for the initiation of pregnancy^49,50^. Myometrial contraction has implications in multi-young species such as rodents for implantation. Once oocytes are fertilized in the oviduct, adrenergic smooth muscle contraction in the uterus is important for the movement of oocytes into the uterine horn. Then, to achieve spatial arrangement of implantation sites, the uterine myometrium assists in transporting the oocytes to the center of the horn. From there, complex contraction dynamics appear to play a role in the spreading of the oocytes along the uterine horn, to provide a spatial arrangement^51^. A future application of this approach is to characterize the electrical activity of the uterus during fertilization, implantation in animal models, throughout pregnancy. In comparison to rodents, humans tend to have singleton pregnancies, but little is known about how the contraction dynamics of the uterine myometrium contribute to sperm and oocyte transport. Observations regarding changes in uterine activity in humans through the menstrual cycle have led to the suggestion that myometrial contractions do influence both oocyte and spermatozoa transport^52^. Future studies in humans to characterize this activity could benefit from consideration of both slow wave and burst activity.

Uterine electrical activity in pregnancy is an area of significant focus in the literature, and the predominant theory indicated a large period of quiescence during pregnancy and coordinated electrical events during late pregnancy and childbirth^1^. The development of novel non-invasive measurement systems for detection of electrical activity during labor shows promising application potential^45,46^, however there remains the challenge of accurately correlating body surface measurements (such as EHG) with the underlying electrical activity occurring at the organ level. This approach can also be applied to non-pregnancy^53^, with the associated challenges of the deep location of the uterus within the abdomen. Measurements obtained directly from the surface of the uterus can be used to help elucidate the relationship between activity at the organ level and those measured on the body surface.

## Conclusions

In conclusion, electrophysiological measurement of the uterine myometrium provides information about contractile activity. The initiation and propagation of electrical activity in the non-pregnant uterus is not well understood, and this novel method of in vivo electrophysiological measurements using high resolution flexible sensors will provide an avenue for investigation. The high frequency and low frequency signals can be isolated and analyzed. We show that, concurrent with the high frequency burst activity previously observed in the non-pregnant uterus, there is a slow wave depolarization which propagates throughout the tissue. Frequency analysis provide information about the dominant frequencies in both the slow wave and the bursts. By selecting a linear array of electrodes in contact with the uterine horn, the direction and velocity of the slow wave as it propagates along the uterine horn can be determined. The novel in vivo approach outlined in this study has the potential to provide the organ level detail to support non-invasive body surface measurement systems and help bridge the organ-to-body-surface knowledge gap.

## Data Availability

The raw data will be made available by the corresponding author on request.
